# Racial and ethnic variation in multigene panel testing in a cohort of *BRCA1/2*‐negative individuals who had genetic testing in a large urban comprehensive cancer center

**DOI:** 10.1002/cam4.4541

**Published:** 2022-01-17

**Authors:** Sushma Tatineni, Meri Tarockoff, Nadine Abdallah, Kristen S. Purrington, Hadeel Assad, Rachel Reagle, Nancie Petrucelli, Michael S. Simon

**Affiliations:** ^1^ Minnesota Oncology Minneapolis Minnesota USA; ^2^ Division of Hematology/Oncology Memorial Healthcare System Pembroke Pines Florida USA; ^3^ Department of Oncology Mayo Clinic Rochester Minnesota USA; ^4^ Department of Oncology Wayne State University School of Medicine Detroit Michigan USA; ^5^ Population Studies and Disparities Research Program Barbara Ann Karmanos Cancer Institute Detroit Michigan USA; ^6^ Invitae Genomics San Francisco California USA

**Keywords:** cancer genetics, cancer prevention, genetic variants, medical genetics

## Abstract

**Background:**

There is sparse clinical information on the racial and ethnic distribution of results of multigene panel testing among individuals at high risk for hereditary cancer.

**Methods:**

We evaluated the results of multigene panel testing across eight clinical sites across the state of Michigan for individuals seen for genetic counseling from May 13, 2013 to October 31, 2019 at the Karmanos Cancer Institute's cancer genetics clinic. We estimated the prevalence of pathogenic variants and variants of uncertain significance (VUS) from genes other than *BRCA1*/*2* among individuals of non‐Hispanic White (NHW), Black or African American (AA), Ashkenazi Jewish (AJ), Arab, Hispanic, and other ancestry.

**Results:**

The racial and ethnic distribution of 2419 individuals who had panel testing included 68.8% NHW, 22.1% AA, 2.3% Arab, 2.2% AJ, 1.0% Hispanic, and 3.6% other. Of these, 11.2% had pathogenic variants and 17.5% had VUS. After multivariable analyses, compared to NHW, AA were less likely to have pathogenic variants (OR 95% CI, 0.38, 0.24–0.59, *p *< 0.001). Both AA and Arabs were more likely to have VUS (OR 95% CI, 1.53, 1.18–1.98, *p *= 0.001 and OR 95% CI, 2.28, 1.17–4.43, *p *= 0.015, respectively). There were no significant differences for other groups. The most common pathogenic variants were *CHEK2* (*n* = 65), *MUTYH* (*n* = 45), *ATM* (*n* = 28), and *MSH2* (*n* = 22); the most common pathogenic variants by race and ethnicity were *CHEK2* (NHW), *MSH2* and *MUTYH* (AA), *MSH2* (Arab), *MSH6* and *CHEK2* (AJ), and *MLH1* (Hispanic); the most common pathogenic variants by primary cancer site were *CHEK2* (breast), *MSH2* (colon), *BRIP1* and *MUTYH* (ovarian), and *MSH2* and *MSH6* (endometrial).

**Conclusions:**

Understanding the racial and ethnic distribution of pathogenic variants in multi‐gene panels has the potential to lead to better identification of individuals at risk for hereditary cancer.

## Graphical abstract

Racial and ethnicity variations were described in patients undergoing multi‐gene panel testing. In addition, common pathogenic variants based on race/ethnicity as well as cancer type was described. This descriptive study allows for informed decisions in counseling of patients.

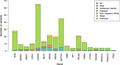

## 1 INTRODUCTION

Recent improvements in next‐generation sequencing (NGS) have revolutionized the ability to screen and test individuals at high risk for hereditary cancer syndromes.^1^ NGS has allowed for the expansion of the scope of genetic counseling and testing and has led to rapid and cost‐efficient assessment of established panels of genes associated with the risk of hereditary cancer.^2^, ^3^

The advent of multiple genetic test panels has resulted in the identification of new pathogenic variants which have potential therapeutic and prognostic value, and has resulted in a large increase in the identification of VUS. Prior studies have evaluated the results of multiple‐gene sequencing panels and have identified the need for additional evaluation assessing clinical utility especially in regards to cancer risk and screening for carriers.^4^, ^5^ In contrast, the identification of VUS may lead to uncertainty in medical management as well as the possibility for increased anxiety and psychosocial stress.^6^

There are established large clinical series documenting the variation in pathogenic and VUS by race and ethnicity among individuals who have had *BRCA1* and *BRCA2* testing,^4^, ^7^ but given the more recent inclusion of genetic test panels for hereditary cancer syndromes, there is less information on racial and ethnic differences in multigene panel results.^8^, ^9^ We present the results of multigene panel testing among individuals who tested negative for *BRCA1*/*2* in a multi‐ethnic, large, urban comprehensive cancer center genetics clinic including individuals seen in both urban and other sites across the state of Michigan. Our aim was to investigate racial and ethnic differences in test results and to describe clinical characteristics in regards to personal and family cancer history.

## 2 METHODS

### 2.1 Study population

Data were collected on individuals who were evaluated for hereditary cancer risk at the Karmanos Cancer Institute (KCI) Cancer Genetic Counseling Service (CGCS) from May 13, 2013 to October 31, 2019. The KCI is a National Cancer Institute designated Comprehensive Cancer Center and is located in Detroit, Michigan. It is affiliated with a network of 14 community sites across Michigan, which together make up the McLaren Medical Network. Individuals were evaluated by the CGCS in Detroit and in seven other McLaren community sites and were offered genetic testing if they met criteria based on National Comprehensive Cancer Network (NCCN) guidelines.^3^ We reviewed genetic test results and clinical information on individuals who tested negative for *BRCA1* and *BRCA2* genes and who also had multigene panel testing. If multiple members of a single family were tested, only the first individual within a family to undergo genetic testing was included in this report. All clinical information was stored in a secure electronic database. Institutional Review Board (IRB) approval was obtained to perform a retrospective review of the prospectively maintained database.

### 2.2 Clinical variables

All participants in the CGCS had a standard three generation pedigree including information on family cancer history and age at diagnosis when available. Our sample included both affected and unaffected individuals. For affected individuals we collected information on the primary cancer site, histologic type, hormone receptor and HER2neu results (for breast cancer), age at cancer diagnosis and genetic testing, gender, and race/ethnicity. Race and ethnic groups were based on self‐identified race and ethnicity and included non‐Hispanic White (NHW), Black or African American (AA), Ashkenazi Jewish (AJ), Arab, Hispanic and other (Asian, Native‐American, and Mixed Race). There were 14 individuals with unknown race and ethnicity information.

### 2.3 Gene testing and sequencing

Genetic testing was conducted at one of three Clinical Laboratories Improvement Amendments (CLIA)‐approved laboratories. All individuals in the study sample had NGS testing of a panel of genes in addition to *BRC*A1 and *BRCA2*. The panels used included varying numbers of genes depending on the date of testing and the pattern of cancer in the family. While all panels were not the same, the genes in the panels included some or all of the following genes in addition to *BRCA1* and *BRCA2*: *MLH1*, *MSH2*, *MSH6*, *PMS2*, *CDH1*, *PTEN*, *STK11*, *ATM*, *VHL*, *BMPR1A*, *CHEK2*, *NBN*, *PALB2*, *MRE11A*, *NF1*, *BARD1*, *RAD51C*, *RAD50*, *BRIP1*, *MUTYH*, *RAD51D*, *POLE*, *TP53*, *DICER1*, *TSC2*, *RECQL4*, *PDGFRA*, and *AXIN2*. Various gene panels used are listed in Table S3.

Comprehensive germline testing and genetic variants were reported through NGS testing based on the standards and guidelines of the American College of Medical Genetics and the Association for Molecular Pathology.^10^ Using these guidelines, variants were classified as pathogenic, likely pathogenic, VUS, benign, or likely benign.^10^, ^11^ In this report, we include test results for pathogenic variants (including likely pathogenic) and VUS in genes excluding results on *BRCA1 and BRCA2*.

### 2.4 Statistical analysis

Distributions of demographic and clinical characteristics of the participants were described using frequency tables and percentages, stratified by variant status as well as by race and ethnicity. We also evaluated differences in the number of times each gene was tested by race and ethnicity using the chi‐squared tests. Multinomial logistic regression was used to estimate odds ratios (ORs) and 95% confidence intervals (CIs) for associations between race and ethnicity and variant status. Multivariable models were adjusted for the potential confounders of age at testing, self‐reported gender, personal history of cancer (yes vs. no), and name of testing panel used. For the race and ethnic group analysis, we included individuals self‐identified as NHW, AA, Hispanic, Arab and AJ and excluded individuals from other groups including Asian, Native‐American, Mixed Race, and Unknown due to small sample sizes. All data were analyzed using R statistical software (https://cran.r‐project.org/). All statistical tests were two‐sided, with a *p* value of <0.05 considered to be statistically significant.

## 3 RESULTS

### 3.1 Participant characteristics and multigene panel testing

There were 3544 individuals evaluated in the CGCS for the risk of hereditary disease across eight clinic sites from May 13, 2013 to October 31, 2019, of which 2433 (68.6%) individuals underwent panel testing. Race and ethnicity data were unavailable for 14 patients, and the analytic cohort was based on 2419 patients. Of those tested, 271 (11.2%) were found to have a pathogenic variant (13 had two variants), 423 (17.5%) had VUS, and 1725 (71.3%) had no pathogenic variant (wild type).

Table 1 shows the demographic, clinical, and family cancer patterns of 2419 individuals who had genetic testing, stratified by race and ethnicity. The cohort was predominantly female (86.9%) and 58% were above the age of 50. There were 1665 (68.8%) NHW, 535 (22.1%) AA, 55 (2.3%) Arab, 53 (2.2%) AJ, 25 (1.0%) Hispanic, and 86 (3.6%) other. Of those tested, there were 1472 (60.9%) affected with cancer, including 1142 (47.2%) with cancers associated with hereditary breast and ovarian cancer syndrome (HBOC). The distribution of those tested by cancer type included 922 (38.1%) with breast; 199 (8.2%) with colon; 131(5.4%) with ovarian; 108 (4.5%) with endometrial; 53 (2.2%) with pancreatic; and 39 (1.6%) with prostate cancer. Other cancer types included skin (29), thyroid (29), gastric (20), renal (19), and bladder (2).

**TABLE 1:** Demographic and clinical characteristics of individuals who had panel genetic testing by race/ethnicity in the Karmanos cancer institute cancer genetics cohort (*n* = 2419)

	AA *N* = 535		Arab *N* = 55		Ashkenazi *N* = 53		Hispanic *N* = 25		NHW *N* = 1665		Other *N* = 86		Total *N* = 2419	
	*n*	%	*n*	%	*n*	%	*n*	%	*n*	%	*n*	%	*n*	%
Variant														
No mutation	388	72.5	30	54.5	44	83.0	18	72.0	1180	70.9	65	75.6	1725	71.3
Pathogenic	26	4.9	11	20.0	4	7.5	1	4.0	221	13.3	8	9.3	271	11.2
VUS	121	22.6	14	25.5	5	9.4	6	24.0	264	15.9	13	15.1	423	17.5
Age at testing														
<50	242	45.2	26	47.3	7	13.2	12	48.0	634	38.1	45	52.3	966	39.9
50 or older	282	52.7	29	52.7	44	83.0	11	44.0	998	59.9	40	46.5	1404	58.0
Unknown	11	2.1	0	0	2	3.8	2	8.0	33	2.0	1	1.2	49	2.0
Gender														
Male	76	14.2	5	9.1	3	5.7	2	8.0	213	12.8	18	20.9	317	13.1
Female	459	85.8	50	90.9	50	94.3	23	92.0	1452	87.2	68	79.1	2102	86.9
Personal cancer history														
None	189	35.3	19	34.5	20	37.7	11	44.0	668	40.1	40	46.5	947	39.1
HBOC	299	55.9	30	54.5	19	35.8	14	56.0	737	44.3	43	50.0	1142	47.2
Breast	263	49.2	27	49.1	11	20.8	12	48.0	574	34.5	35	40.7	922	38.1
HR^+^/HER2^−a^	63	24.0	7	25.9	3	27.3	4	33.3	147	28.0	8	22.9	232	9.6
HR^+^/HER2^+a^	26	9.9	4	14.8	0	0	0	0	56	10.7	6	17.1	92	3.8
HR^−^/HER2^+a^	7	2.7	1	3.7	0	0	0	0	29	5.5	2	5.7	39	1.6
Triple Neg ^a^	46	17.5	2	7.4	5	45.5	0	0	49	9.3	4	11.4	106	4.4
HR^+^/HER2 unknown^a^	88	33.5	14	51.9	1	9.1	4	33.3	194	37.0	10	28.6	311	12.9
HR‐/HER2 unknown ^a^	26	9.9	1	3.7	0	0	3	25.0	47	9.0	5	14.3	82	3.4
Breast <age 45 ^a^	87	33.1	7	25.9	1	9.1	3	25.0	114	21.7	7	20.0	219	9.1
Colon	56	10.5	5	9.1	1	1.9	1	4.0	128	7.7	8	9.3	199	8.2
Ovarian	22	4.1	3	5.5	5	9.4	1	4.0	95	5.7	5	5.8	131	5.4
Endometrial	20	3.7	2	3.6	4	7.5	1	4.0	78	4.7	3	3.5	108	4.5
Thyroid	1	0.2	0	0	0	0	0	0	26	1.6	2	2.3	29	1.2
Renal	2	0.4	0	0	1	1.9	0	0	16	1.0	0	0	19	0.8
Gastric	6	1.1	2	3.6	0	0	0	0	12	0.7	0	0	20	0.8
Skin	0	0	0	0	1	1.9	1	4.0	27	1.6	0	0	29	1.2
Pancreas	9	1.7	0	0	5	9.4	0	0	36	2.2	3	3.5	53	2.2
Bladder	1	0.2	0	0	0	0	0	0	1	0.06	0	0	2	0.1
Prostate	12	2.2	0	0	0	0	0	0	27	1.6	0	0	39	1.6
Family cancer history														
HBOC^b^	319	80.0	24	72.7	37	84.1	7	46.7	869	80.6	42	72.4	1298	53.7
Breast	255	63.8	20	60.6	29	65.9	7	46.7	678	62.9	30	51.7	1019	42.1
Breast ca <age 50	130	32.5	13	39.4	12	27.3	2	13.3	284	26.3	13	22.4	454	18.8
Ovarian cancer	60	15.0	6	18.2	4	9.1	1	6.7	182	16.9	11	19.0	264	10.9

Abbreviation: NHW, non‐Hispanic white.

^a^ Percentages are based on race‐specific total numbers of participants with a personal history of breast cancer.

^b^ Cancers associated with hereditary breast and ovarian cancer syndrome including breast, ovarian, pancreatic, prostate and melanoma.

A relatively higher percentage of Arabs (20%) was noted to have pathogenic variants in comparison to the other groups and higher relative rates of VUS among those who were AA, Arab, and Hispanic. In all race and ethnic groups, HBOC was the most common cancer type. The most common subtype of breast cancer in AA, Arab, Hispanic, and NHW groups was HR + although HER2 status was unknown in 16.3% of cases. Triple‐negative breast cancer was most predominant in AJ (45.5%). The highest percentage of breast cancer less than age 45 was seen in AA (33.1%) followed by Arabs (25.9%). In terms of family history, Arabs had the highest rate of breast cancer in family members who were less than age 50 at diagnosis (39.4%). Similar rates of family history of breast cancer were seen in AA, Arab, AJ, and NHW. There were 84.1% of AJ and 80% of AA and NHW with a family history of HBOC‐related cancer.

As demonstrated in Table S1, there was a roughly equal distribution of pathogenic variants and VUS across age groups and gender. For the majority of affected individuals, there was a higher proportion of individuals with VUS per cancer site compared to the proportion with a pathogenic variant, except for colorectal cancer (CRC) and endometrial cancer for which there was a greater proportion of individuals with a pathogenic variant compared to VUS; 17.8% versus 12.4% and 16.7% versus 14.8%, respectively. By breast cancer subtype, the highest rate of pathogenic variants was seen for women with HR‐ HER2+ (12.8%) cancers, although this was based on small numbers, followed by triple‐negative (11.3%). The rate of pathogenic variants among individuals with ovarian cancer was 9.9%.

### 3.2 Pathogenic variants and VUS by race and ethnicity

Given the variability of gene panels tested by race and ethnicity, chi‐squared testing was performed to assess the difference in proportion of individuals tested for each gene by race or ethnic group (Table S2). There were significant differences in the frequency of testing for genes including MUTYH, MYH, MLH1, and MSH2 by race and ethnicity. In order to account for this in the analyses, the testing panel used was included as a covariate in the adjusted logistic regression analyses. Table 2A,B presents the results of multinomial logistic regression comparing the likelihood of pathogenic variants and VUS, respectively, by race and ethnicity using NHW as the reference group. AA individuals were less likely to carry pathogenic variants than NHW (OR 95% CI, 0.38, 0.24–0.59, *p *< 0.001) and both AA and Arabs were more likely to have VUS (OR 95% CI, 1.53, 1.18–1.98, *p *= 0.001 and OR 95% CI, 2.28, 1.17–4.43, *p *= 0.015, respectively). There were no significant differences in the prevalence of pathogenic or VUS for other racial or ethnic groups compared to NHW.

**TABLE 2:** Results of multinomial logistic regression analyses comparing the rate of (A) pathogenic and (B) VUS results by race and ethnicity in the Karmanos cancer institute cancer genetics cohort

Race/ethnicity	Pathogenic						
	Unadjusted				Adjusted^b^		
	Odds ratio (OR)	95% CI univariate	*p* Value	OR	95% CI multivariate	*p* value	% of total
(A) Rate of pathogenic variants							
NHW	1	—	—				13.3
Black or African American	0.36	0.23–0.5	<0.001	0.38	0.24–0.59	<0.001	4.9
Arab	1.96	0.97–3.96	0.062	2.00	0.95–4.22	0.069	20.0
Ashkenazi Jewish	0.49	0.17–1.36	0.17	0.46	0.46–1.33	0.15	7.5
Hispanic	0.29	0.04–2.23	0.24	0.32	0.04–2.65	0.29	4.0
Other^a^	0.89	0.49–1.39	0.27	0.66	0.31–1.43	0.30	9.3

	Unadjusted			Adjusted			
	Odds ratio (OR)	95% CI	*p* value	Odds Ratio (OR)	95% CI	*p* value	% of total
(B) Rate of Variants of uncertain significance							
NHW	1	—	—				15.9
Black or African American	1.39	1.09–1.77	0.0076	1.53	1.18–1.98	0.0013	22.6
Arab	2.09	1.09–3.99	0.026	2.28	1.17–4.43	0.015	25.5
Ashkenazi Jewish	0.51	0.20–1.29	0.16	0.56	0.22–1.45	0.23	9.4
Hispanic	1.49	0.59–3.79	0.40	1.68	0.64–4.39	0.29	24.0
Other^a^	0.89	0.49–1.65	0.72	0.98	0.52–1.82	0.94	15.1

Abbreviation: NHW, non‐Hispanic white.

^a^ Other: Asian, Native American, Mixed Race and Unknown.

^b^ Adjusted for age at testing, gender, personal history of cancer, and testing panel.

**FIGURE 1:**
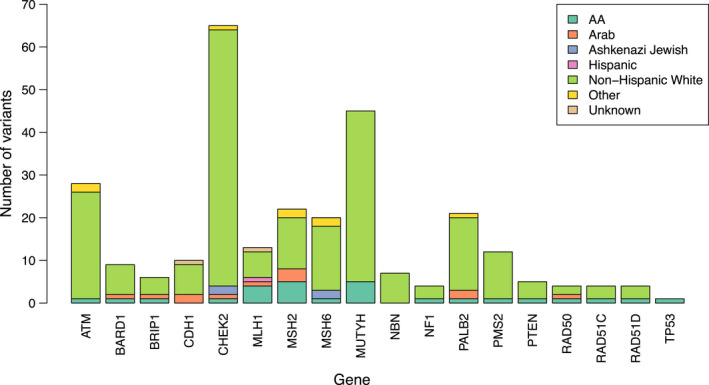
Distribution of race and ethnicity by gene among individuals with a pathogenic variant

Figure 1 shows the distribution of race and ethnic groups by genes identified with pathogenic variants. The most common pathogenic variants were *CHEK2* in NHW, *MSH2* and *MUTYH* in AA, *MSH6* and *CHEK2* in AJ, *MSH2* in Arabs, and *MLH1* in Hispanics. Focusing on the four most common genes for which pathogenic variants were identified, for individuals with a pathogenic variant in *CHEK2*, the race and ethnic breakdown included 92.3% NHW, 1.5% AA, 1.5% Arab, and 3.1% AJ. Among individuals with pathogenic variants in *MUTYH*, 88.9% were NHW and 11.1% were AA. For *ATM*, 89.3% were NHW and for *MSH2*, 54.5% were NHW, 22.7% were AA, and 13.6% were Arab.

For NHW with *CHEK2*, the most common variant was 1100delC, occurring in 13 out of 56 (23.2%) of those with pathogenic variants. For NHW with *ATM*, 5290delC and c.5763–1050>G were each seen in 2 out of 25 (8%) of those with pathogenic variants. For *MUTYH*, the most common variant among NHW was c.1187G>A seen in 8 out of 34 (23.5%) and for *PALB2*, c.3113G>A was seen in 2 out of 16 (12.5%) of the White probands.

For Arabs with *MSH*2, all three individuals had the common variant c.932delA. Among three Arabs with *PALB2* pathogenic variants, two variants were identified (c.1056_1057delGA and p. Arg1086). In AA and AJ probands there were no variants seen more than once.

### 3.3 Pathogenic variants by cancer type

**FIGURE 2:**
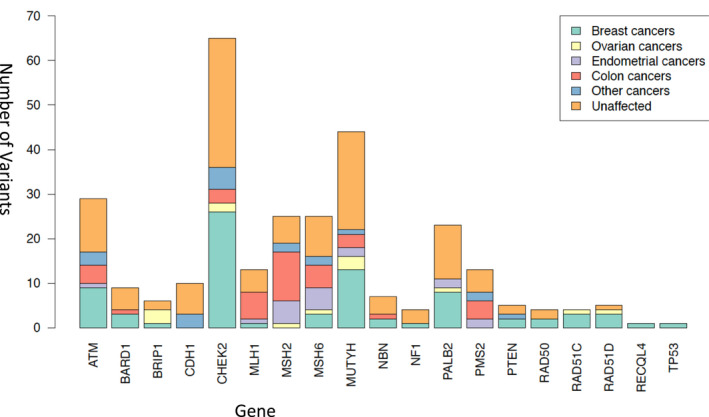
Distribution of primary cancer sites by gene among individuals with a pathogenic variant

Figure 2 lists the distribution of primary cancer types by gene among individuals with pathogenic variants for 2433 individuals who underwent panel testing. The four most common genes for which there were pathogenic variants identified included *CHEK2* (*n* = 65), *MUTYH* (*n* = 45), *ATM* (*n* = 28), and *MSH2* (*n* = 22). Of individuals with a *CHEK2* pathogenic variant, 40% had breast cancer, 3% had ovarian cancer, and 4.6% had colon cancer. Among those with *MUTYH*, 28.9% had breast cancer, 6.7% had ovarian cancer, 4.4% had endometrial cancer, and 6.7% had colon cancer. For those with *ATM* 32% had breast cancer, 3.6% had endometrial cancer, and 14.3% had colon cancer. Lastly for *MSH2*, 5.3% had ovarian cancer, 17.9% had endometrial cancer, and 39.3% had colon cancer.

For 925 probands with a diagnosis of breast cancer, the most commonly occurring pathogenic variant was in *CHEK2* (*n* = 26). Of 12 individuals with triple‐negative breast cancer, the most common pathogenic variant was in *BARD1* (*n* = 2). For 202 probands with colon cancer, the most commonly occurring pathogenic variant was in *MSH2* (*n* = 11) and for 131 women with ovarian cancer, the most common pathogenic mutations were in *BRIP1* (*n* = 3) and *MUTYH* (*n* = 3). For 108 women with endometrial cancer, the most common pathogenic mutations were in *MSH2* (*n *= 5) and *MSH6* (*n* = 5).

### 3.4 Pathogenic variants and family history

The cohort of tested individuals included 1298 (53.7%) with a family history of an HBOC‐related cancer, 1019 (42.1%) with a family history of breast cancer alone, 454 (18.8%) with a family history of breast cancer with at least one individual diagnosed at age less than 50, and 264 (10.9%) with a family history of ovarian cancer (Table 1).

The highest rate of pathogenic variants among these groups was seen among individuals with a family history of ovarian cancer (14.6%). For individuals with a family history of breast cancer and a family history of breast cancer at age <50, there were 9.7% and 9.6% with pathogenic variants, respectively. In those with history of cancers associated with HBOC, 10.6% had pathogenic variants. Of 65 individuals with *CHEK2*, there were 50% with a family history of breast cancer, 34% with a family history of breast cancer at a young age at <age 50, 14.3% with a family history of ovarian cancer, and 21% with other HBOC‐related cancers in their families. Of 28 with *ATM*, 28.6% had a family history of breast cancer, 3.6% had a history of breast cancer at a young age (less than age 50), 7.1% had a family history of ovarian cancer, and 25% had a family history of cancers associated with HBOC (Table S1).

## 4 DISCUSSION

There is now substantial literature on multigene panel testing in individuals at high risk for hereditary disease, and the majority of prior studies suggest benefit over single gene(s) testing in regards to identification of other hereditary syndromes and the potential for screening and cascade testing.^5^, ^8^, ^12^, ^13^ In this study, we focused on the rate of pathogenic variants and VUS among people of different racial and ethnic backgrounds and identified the clinical patterns of those variants by assessing the relationship by cancer type and patterns of cancer in the family. Our goal was to describe differences based on race and ethnicity as well as cancer type. Of 2419 individuals in our testing cohort with available race and ethnicity data, the rate of pathogenic variants and VUS was 11.2% and 17.5%, respectively, which was similar to results reported by others.^5^

Few other studies have described racial and ethnic variation in multi‐panel genetic testing among individuals at high risk for hereditary cancer syndrome. Although the results presented in this paper represent data from a single institution, given the location of our medical facility in Detroit, MI, the presence of an ethnically and racially diverse cohort of patients, and the recruitment of patients from satellite sites across the State of Michigan, allowed us to evaluate the distribution of pathogenic and VUS results among different groups. Based on the 2019 United States census bureau, the racial and ethnic distribution of the population in the United States is 60.1% NHW, 18.5% Hispanic, 13.4% AA, 5.9% Asian, 2.3% Arab, 2.2% AJ, and 1.3% Native American. Michigan in comparison to the US population has a higher proportion of NHW (74.9%), AA (14.1%), and a lesser proportion of Hispanic (5.2%), Asian (3.4%), AJ (0.9%), and an equivalent proportion of Arabs (2.3%), with 12% of the US Arab population residing in the state of Michigan, the majority of whom live in Dearborn, MI.^14^ The race and ethnic breakdown of our cohort however, is a similar cross‐sectional representation of the US population in terms of NHW (68.8%), Arab (2.3%), and AJ (2.2%). Our cohort had a higher percentage of AA individuals (22.1%) and a lesser number of Hispanics (1%). This distribution of individuals in our cohort provided a unique opportunity to assess racial and ethnic differences in genetic test results.

In our database, *CHEK2* was found to have pathogenic variants with an overall prevalence of 2.2% and was the gene most likely associated with a family history of cancer and among NHW patients. Among those with a pathogenic variant in *CHEK*2, there were 50% with a family history of breast cancer in contrast to those with *ATM* where there were only 28.6% with a family history of breast cancer.

In our cohort, the most common specific *CHEK2* variant was 1100delC which has been reported to be associated with increased risk of death from breast cancer, increased risk of male breast cancer, and an increased risk of second breast cancer^15^, ^16^as well as an increased risk of estrogen receptor (ER)‐positive breast cancer.^15^, ^17^ The *CHEK2* 1100delC is also the only *CHEK2* allelic variant known to be associated with breast cancer risk factors as well as early death from breast cancer, ER‐positive breast cancer, and a diagnosis of a second breast primary.^18^ Other pathogenic *CHEK2* variants noted in our database included p.I157T and c.444+1G>A, which have also been seen by others,^19^ although cancer risks associated with many of these variants are not known.^18^ CHEK2 was predominantly seen in NHW, with few occurrences in other race and ethnic groups, although sample sizes in our cohort for these groups were small. Our results are similar to data outlined in previous studies.^20^, ^21^

As has been shown in reports of *BRCA1*/*2*VUS,^22^ AA individuals in our dataset were more likely to have VUS as were people of Arab ancestry. The most common pathogenic variants seen were *MUTYH* and *MSH2* in AA and *MSH2* in Arabs. When compared within groups, Arabs had the highest rate of pathogenic variants (20.0%) out of all race/ethnic groups, but these results were not statistically significant on multivariate analysis adjusted for variation in gene panels tested and other factors predictive of genetic testing.

Given the heterogenicity in genetic test results across race and ethnic groups, it is important to consider panel genetic testing across all groups of high‐risk individuals. Specifically, in Arabs, the most common variants in our sample were in the *MSH2* and *PALB2* genes. Of note, all three Arab individuals with a pathogenic variant in *MSH2* had the c.932delA variant, and both Arab individuals with a pathogenic variant in *PALB2* had the c.1056_1057delGA variant. To the best of our knowledge, these individuals were unrelated. Review of other studies which described the frequency of pathogenic variants in subsets of the Arabic populations have, to the best of our knowledge, not described these specific variants.^23^, ^24^ Further larger series evaluating the types of pathogenic variants and cancer types among different race and ethnic groups would provide more information on the importance of the pathogenic variants described especially in our patients with Arab ethnicity.

In our cohort of women with breast cancer, the most common pathogenic variant was found in the *CHEK2* gene and among individuals with colon cancer, the most common pathogenic variant was found in the *MSH2* gene. Prior studies have described an association of TNBC and pathogenic variants in *BARD1* ^25^, ^26^; among 12 TNBC patients with pathogenic variants in our cohort, two (16.7%) had pathogenic variants in *BARD1*. However, the frequency of *BARD1* observed in our cohort among all pathogenic variants was 3.3% and we cannot make any conclusions about the association of TNBC with *BARD1*. Other pathogenic variants seen in our cohort of TNBC patients included two with *RAD51C* and one each with *ATM*, *PALB2*, *PTEN*, *POLE*, *PDGFRA*, and *MUTYH* genes. In individuals with a family history of breast cancer, young onset breast cancer, and ovarian cancer, the most common pathogenic variant was in *CHEK2*.

The use of genetic testing has increased dramatically since the completion of the human genome project in 2003, and in 2013, the supreme court invalidated patenting of genetic testing for *BRCA1* and *BRCA2*,^27^ which resulted in a change in genetic testing patterns where now the majority of commercial laboratories offer testing of multiple panels of genes using NGS^5 1^. The use of panel gene testing is also supported by lower costs of testing^5^; however, questions arise as to the necessity and benefits of multigene panel testing, given the lack of established clinical utility for many pathogenic variants identified. In addition, further complexity arises with the increased identification of VUS^28^ in regards to medical management and patient anxiety,^6^ although other reports have suggested that women did not have an increase in psychosocial problems following genetic testing.^29^ We identified a higher percentage of pathogenic variants (20.0%) among the Arab population in comparison to NHW (13.3%) although there was a measurable difference in population sizes in these two cohorts. However, similar to prior studies,^21^ we did not identify a statistically significant higher rate of pathogenic variants across different race and ethnic groups on multivariate analysis. Our data also demonstrate greater VUS in AA and Arabs, similar to observations made that with increased gene sequencing over time, a greater number of VUS is seen across different racial and ethnic groups.^21^, ^30^

Strengths of our analysis include the large heterogeneous database with a population distribution similar to the US population with information on personal cancer history, three generations of family cancer history, and testing in CLIA‐approved laboratories. We also had an equivalent number of Arabs and AJ and a relatively higher proportion of AA individuals in comparison to the general population. Limitations included the fact that the study sample was derived from a genetics clinic affiliated with an NCI sponsored comprehensive cancer center thereby attracting mostly high‐risk individuals with results that are possibly not generalizable to other settings, leading to possible sample selection bias. In addition, although our description of unique findings in the various racial and ethnic groups, including the Arabic population has not been well described in prior studies, our population numbers were still small and further studies with larger populations would be beneficial for more accurate interpretations. In regards to patterns of genetic test results by race and ethnicity, other potential confounders of testing including gene panel used, age at testing, gender, and personal history of malignancy were included; however, there is also the possibility of selection bias based on which individuals seek consultation or who are referred to a cancer genetics clinic. Our study population also included mostly individuals with a diagnosis of breast cancer or a family history of breast cancer or other HBOC‐related cancers, and a better understanding of the variation in panel genetic test results could be derived from population samples with larger number of other cancer types.

In conclusion, results from our cohort demonstrate the diversity in genetic test results by racial and ethnic groups and confirm the importance of continued panel genetic testing. These findings support the need to further address studies of various race and ethnic groups to identify unique pathogenic variants and further re‐classify VUS, allowing us to better identify individuals at high risk for hereditary cancer across all race and ethnic groups. Access to genetic testing for all groups of high‐risk individuals and understanding the prevalence of variants in multigene panels can lead to better identification of individuals at high risk for hereditary cancer across various ethnic groups, who can benefit from enhanced surveillance and risk‐reducing management.

## CONFLICT OF INTEREST

There is no conflict of interest.

## AUTHOR CONTRIBUTION

All authors mentioned in the manuscript have agreed for authorship, read and approved the manuscript, and given consent for submission and subsequent publication of the manuscript.

## Supporting information

Table S1

Table S2

Table S3

## Data Availability

Detailed data and analysis are available upon request.
