# Supplementary material for: Racial and ethnic variation in multigene panel testing in a cohort of BRCA1/2‐negative individuals who had genetic testing in a large urban comprehensive cancer center
Source: Cancer Med. 2022 Jan 17;11(6):1465–73. doi: 10.1002/cam4.4541 (PMC8921894; doi:10.1002/cam4.4541)
Supplement: Supplementary file 1 — Table S1 [file CAM4-11-1465-s003.pdf]

Supplemental Table 1:
Demographic and Clinical Characteristics of Individuals who Underwent Panel Genetic Testing by Genetic Test Result

Characteristic		No mutation/ benign (n=1736)	%	Pathogenic (n=273)	%	VUS (n=424)	%
**Age at testing**							
<50		704	72.4	106	10.9	162	16.7
50 or older		995	70.5	160	11.3	257	18.2
Unknown		43	87.8	3	6.1	3	6.1
**Gender**							
Male		234	73.6	33	10.4	51	16.0
Female		1499	71.1	238	11.3	372	17.6
Unknown		3	50.0	2	33.3	1	16.7
**Personal Cancer History**							
None		678	70.6	117	12.2	166	17.3
HBOC [1]		834	72.7	98	8.5	215	18.7
Breast		675	73.0	78	8.4	172	18.6
	HR+/HER2-	28	73.3	5	8.2	6	18.5
	HR+/HER2+	71	76.3	9	9.7	13	14.0
	HR-/HER2+	28	771.8	5	12.8	6	15.4
	Triple Neg	82	77.4	12	11.3	12	11.3
	HR+/HER2 unknown	210	67.5	29	9.3	72	23.2
	HR-/HER2 unknown	63	76.8	6	7.3	13	15.9
Breast <45		175	79.2	16	7.2	30	13.6
Colon		141	69.8	36	17.8	25	12.4
Ovarian		94	71.8	13	9.9	24	18.3
Endometrial		74	68.5	18	16.7	16	14.8
Thyroid		16	55.2	3	10.3	10	34.5
Renal		15	78.9	2	10.5	2	10.5

Gastric	14	70.0	3	15.0	3	15.0
Skin	19	61.3	5	16.1	7	22.6
Pancreas	39	`73.6	1	1.9	13	24.5
Bladder	1	50.0	1	50.0	0	0
Prostate	28	71.8	3	7.7	8	20.5
**Family Cancer History**						
HBOC [2]	950	72.6	139	10.6	220	16.8
Breast	753	73.2	100	9.7	175	17.0
Breast ca <age 50	333	72.7	44	9.6	81	17.7
Ovarian Cancer	186	69.7	39	14.6	42	15.7

1. NHW – Non-Hispanic White
2. Cancers associated with hereditary breast and ovarian cancer syndrome including breast, ovarian, pancreatic, prostate and melanoma.
