# Supplementary material for: Racial and ethnic variation in multigene panel testing in a cohort of BRCA1/2‐negative individuals who had genetic testing in a large urban comprehensive cancer center
Source: Cancer Med. 2022 Jan 17;11(6):1465–73. doi: 10.1002/cam4.4541 (PMC8921894; doi:10.1002/cam4.4541)
Supplement: Supplementary file 2 — Table S2 [file CAM4-11-1465-s002.pdf]

Supplemental Table 2:

Gene	p-value*	Number of patients tested for each gene					
		AA	Arab	Ashkenazi	Hispanic	NHW	Other
ATM	0.63269243	455	45	42	23	1430	73
BARD1	0.52340535	383	37	32	19	1168	57
BRIP1	0.58841279	437	44	40	23	1331	69
CDH1	0.34116859	475	50	42	23	1485	76
CHEK2	0.61932124	493	50	45	23	1508	78
MSH6	0.08389936	308	32	33	15	1066	47
MUTYH	0.02341501	422	39	37	20	1189	60
MYH	0.02341501	422	39	37	20	1189	60
NBN	0.45453736	386	37	32	19	1175	57
NF1	0.3521172	379	36	32	19	1111	56
PALB2	0.39397783	460	46	42	23	1460	74
PMS2	0.06126527	367	40	41	19	1257	62
PTEN	0.07475012	446	43	36	19	1343	65
RAD50	0.05567213	406	39	35	19	1147	58
RAD51C	0.2208421	373	35	30	19	1094	54
RAD51D	0.06200092	405	38	34	19	1152	57
TP53	0.11605256	412	41	32	18	1211	65
MLH1	0.00629641	278	30	28	14	1016	45
MSH2	0.00178225	273	29	28	14	1011	43

*Chi-squared test testing difference in proportion of patients tested for each gene by race
