# Supplementary material for: Racial and ethnic variation in multigene panel testing in a cohort of BRCA1/2‐negative individuals who had genetic testing in a large urban comprehensive cancer center
Source: Cancer Med. 2022 Jan 17;11(6):1465–73. doi: 10.1002/cam4.4541 (PMC8921894; doi:10.1002/cam4.4541)
Supplement: Supplementary file 3 — Table S3 [file CAM4-11-1465-s001.pdf]

Supplemental Table 3: List of Gene Panel Tests

Comprehensive COLARISÆ
ColoNextÆ
OvaNextÆ
CancerNextÆ
PancNext
BROCA
GYNplus
RenalNext
Comprehensive Cancer Panel (Gene Dx)
Comprehensive Lynch Gene Panel
Hereditary Colon Cancer Panel
ProstateNext
Invitae Breast and Gyn Panel
Lynch Syndrome Panel
Invitae Common Cancer Panel
Quest Panel
TumorNext
Invitae Pancreas Cancer Panel
Quest MyVantage Cancer Panel
Invitae Renal Panel
Expanded-CustomNext
Expanded-CancerNext
Invitae Multi Cancer Panel
BreastNextÆ
BRCAplus
myRisk
High Risk Hereditary Breast Cancer
Hereditary Breast Cancer Extended Panel
CustomNext
Counsyl Comprehensive Panel
Invitae Familial GIST Panel
Invitae PCC/PGL Panel
Invitae Breast STAT Panel
Invitae Breast Cancer Panel
CustomNext-Cancer
